# Analysis of Total-Forms of Cyanotoxins Microcystins in Biological Matrices: A Methodological Review

**DOI:** 10.3390/toxins14080550

**Published:** 2022-08-11

**Authors:** Pierre Bouteiller, Emilie Lance, Thierry Guérin, Ronel Biré

**Affiliations:** 1 Université de Reims Champagne-Ardenne, UMR-I 02 INERIS-URCA-ULH SEBIO, Unité Stress Environnementaux et BIOsurveillance des Milieux Aquatiques (SEBIO), BP 1039, CEDEX 2, F-51687 Reims, France; 2 Laboratory for Food Safety, ANSES, F-94701 Maisons-Alfort, France; 3 UMR MNHN/CNRS MCAM, Muséum National d’Histoire Naturelle, F-75005 Paris, France; 4 Strategy and Programs Department, ANSES, F-94701 Maisons-Alfort, France

**Keywords:** cyanotoxins, microcystins, total toxins content, method of analysis, MMPB method, ozonolysis, deconjugation at basic pH, laser irradiation desorption

## Abstract

Microcystins (MCs) are cyclic heptapeptidic toxins produced by many cyanobacteria. Microcystins can be accumulated in various matrices in two forms: a free cellular fraction and a covalently protein-bound form. To detect and quantify the concentration of microcystins, a panel of techniques on various matrices (water, sediments, and animal tissues) is available. The analysis of MCs can concern the free or the total (free plus covalently bound) fractions. Free-form analyses of MCs are the most common and easiest to detect, whereas total-form analyses are much less frequent and more complex to achieve. The objective of this review is to summarize the different methods of extraction and analysis that have been developed for total forms. Four extraction methods were identified: MMPB (2-methyl-3-methoxy-4-phenylbutyric acid) method, deconjugation at basic pH, ozonolysis, and laser irradiation desorption. The study of the bibliography on the methods of extraction and analysis of the total forms of MCs showed that the reference method for the subject remains the MMPB method even if alternative methods and, in particular, deconjugation at basic pH, showed results encouraging the continuation of the methodological development on different matrices and on naturally-contaminated samples.

## 1. Introduction

Cyanobacteria are photosynthetic microorganisms that appeared on Earth 3.5 billion years ago and can be observed in various freshwater, brackish and marine ecosystems [1]. In freshwater ecosystems, cyanobacteria have significant proliferative capacities [2] favored by different natural factors (e.g., temperature above 25 °C, alkaline pH) but also anthropogenic factors (e.g., high nitrate and phosphorus inputs) [3,4,5]. Many toxins can be produced by cyanobacteria during blooms, such as neurotoxic anatoxins and hepatotoxic microcystins (MCs). The hepatotoxin MCs are the most frequently detected cyanotoxins in cyanobacterial blooms [2]. There is a great diversity of MCs-producing strains of cyanobacteria. MC producers can be found in several different orders, including *Chroococcales*, *Oscillatoriales*, *Nostocales*, and *Stigonematales*. Within these orders, it is important to note that not all genera of an order produce MCs (for example, in the order *Nostocales*, some species of the genus *Anabaena* are producers while other genera of this order, such as *Aphanizomenon*, have never been described as MC-producers). Moreover, a species can be both producing and non-producing depending on the strain, and a producing strain depends on environmental factors [6]. Among the genera known to include MC-producing (and non-producing) species: *Microcystis*, *Planktothrix*, *Anabaena*, and *Hapalosiphon* are the main ones. However, there are many other producing species (Table S1, [6,7]).

**Figure 1:**
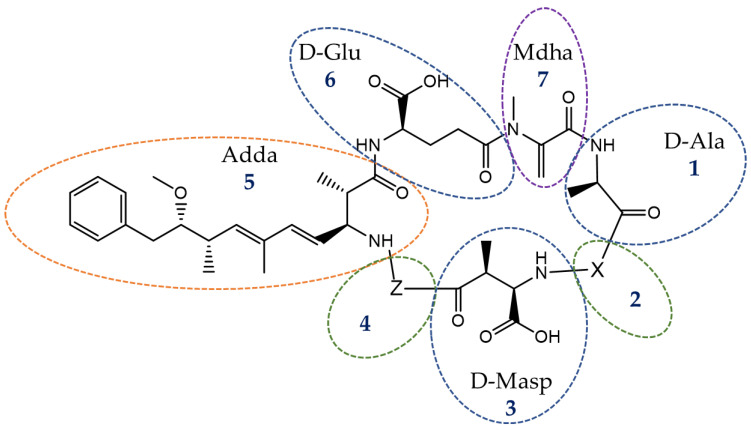
Topological representation of an MC with 1: D-Alanine, 2: X (variable), 3: D-erythro-b-methyl-D-aspartic acid, 4: Z (variable), 5: Adda, 6: D-Glutamate, 7: Mdha. The Adda fragment is a common part of all MC variants and nodularins, another cyanotoxins. The Mdha fragment plays a role in the binding of MCs to proteins. The variable amino acids (X and Z) as well as the possibility of substitution on 1, 3, 5, 6, 7 will define the identity of the MCs variant and participates in the diversity of the MCs. 279 MCs congeners have been listed in Bouaïcha et al. [9].

MCs are monocyclic heptapeptides containing two atypical amino acids called Adda (3-amino-9-methyoxy-2,6,8-trimethyl-10-phenyl-4,6-decadienoic acid), which is a fragment common to all MCs variant and nodularins and Mdha (N-methyl-dehydroalanine) groups, which plays a role in the binding of MCs to proteins. MCs present with two variable amino acids, characteristic of the identity of the MC variant (Figure 1). For example, MC-LR, the first chemically-identified MC, is a variant where L and R represent the amino-acid leucine and arginine, respectively [8]. Thus, the identification of the MCs is mainly due to the variations in the AAs in positions X and Z (Figure 1) but also due to the variations of the other sites of the MCs, which can also be subject to substitutions. To date, up to 279 MCs congeners have been described [9]. MCs are mainly intracellular during cyanobacteria proliferation and are released in media, becoming extracellular (dissolved in water or adsorbed on particles) once cell death occurs after bloom collapse. After the ingestion of cyanobacterial cells or the ingestion of contaminated water, MCs can accumulate in various organisms, such as zooplankton, zoobenthos, or fish, with a possible transfer between trophic levels [10,11,12,13]. MCs can accumulate in two forms in organisms: a free, available cellular fraction and a bound form (*id.* intracellular for the cyanobacterial matrices or covalently bound to proteins in biota tissues). These covalently-bound forms are particularly bound to the serine/threonine residues of enzyme protein phosphatases 1 (PP1) and 2A (PP2A) [14]. The mechanism of the binding of MCs to PP is a two-step mechanism, first with the formation of a weakly-interacting bond of a hydrophobic and reversible nature between the Mdha group of MCs and the proteins and, after a few hours, the Adda group can form a strong covalent thioether bond with the cysteine residues present at the catalytic subunits of PP [15,16,17,18]. The binding of MCs to proteins results in the deregulation of the PP by disrupting the phosphorylation mechanisms [19,20]. This deregulation by MCs may induce a cascade of negative side effects, in particular, tumor promotion and apoptosis, but also the production of reactive oxygen species, resulting in oxidative stress (reviewed in [20,21]). MC toxicity can occur in different organs but was initially discovered in the liver, resulting in the classification of MCs as hepatotoxins. Other organs may be targeted by MCs, including the reproductive organs, kidneys, brain, heart, gastrointestinal tract, or lungs (reviewed in [22]). This specific organo-toxicity can be explained by the tropism of MC towards organs containing organic-anion-transporting polypeptides (OATPs) [23]. The health impact of these toxins is therefore not marginal, and cases of human and animal poisoning are regularly reported (reviewed in [24]). These acute or chronic intoxications can occur after swimming in contaminated environments or after the ingestion of contaminated drinking water or aquatic organisms and cause symptoms of varying intensity ranging from gastroenteritis to kidney and liver failure, sometimes even death (reviewed in [25]).

For human and environmental health risk assessments, either cyanobacteria or intracellular and extracellular cyanotoxins can be monitored from water samples. The monitoring of cyanobacterial density or cyanotoxin concentration limits the exposure of the population to cyanotoxins, particularly during swimming activities, but also in the case of the consumption of species that may bioaccumulate these toxins. For this purpose, a panel of techniques is available for the analysis of cyanotoxins in various matrices (water, sediments, and animal tissues). More specifically, the analysis of MCs can concern the free or the total (free plus covalently bound) fraction. The analyses of free forms of MCs are the most widespread and the simplest to perform (reviewed in [26]). These analyses generally consist of an extraction step with different mixtures of organic solvents (methanol, acetonitrile, butanol, acetone, water, chloroform, hexane, and ethanol) that are either pure or diluted according to the protocols. These extraction steps are sometimes followed by a purification step. Three main methods are described in the literature: liquid–liquid extraction (LLE) [27], solid-phase-extraction (SPE) [28,29,30], or the use of immunoaffinity columns (IAC) [31,32,33,34].

The extracts obtained from the extraction and purification steps can be analyzed by various methods, including immunological, chemical, or biological methods. ELISA (enzyme-linked immunosorbent assay) tests are the most common immunological methods [35,36], although other approaches exist, such as aptamers [37,38,39,40] or biosensors (reviewed in [8]). Chemical approaches are also widely used by using equipment including high-pressure liquid chromatography (HPLC) coupled with a diode array detector (HPLC-UV-DAD) [41,42,43] or capillary electrophoresis [44,45,46,47,48]. However, the most used and sensitive chemical method remains liquid (LC-MS) or gas (GC-MS) chromatography coupled with mass spectrometry [29,49]. The biological approach, including bioassays on rodents [50], plants and algae [51,52,53], invertebrates [54,55], fish and amphibians [56], or in vitro bioassays [57] seems to be less and less used. The protein phosphatase inhibition assay (PPIA), which measures the inhibitory effect of MCs on the activity of PP, is still quite widespread and can be very useful for screening MCs, particularly when coupled with a chemical method such as LC-MS [58]. Complementary to these methods of analysis of free toxins, methods are available to analyze both free and bound (i.e., total) MCs in biological matrices (cyanobacteria or animal tissues). These methods of analysis of the total forms of MCs are still rarely applied as they are more complex to use than the methods of analysis of the free forms of MCs. However, the bound forms can account for the majority of the MCs in a given sample [59,60]. The aim of this literature review is, therefore, to give an overview of existing techniques for the analysis of total forms of MCs. Sample preparation methods (extraction and purification), analysis methods, and potential matrix effects will be reported.

## 2. Quantification of Total MC by the MMPB Method

**Figure 2:**
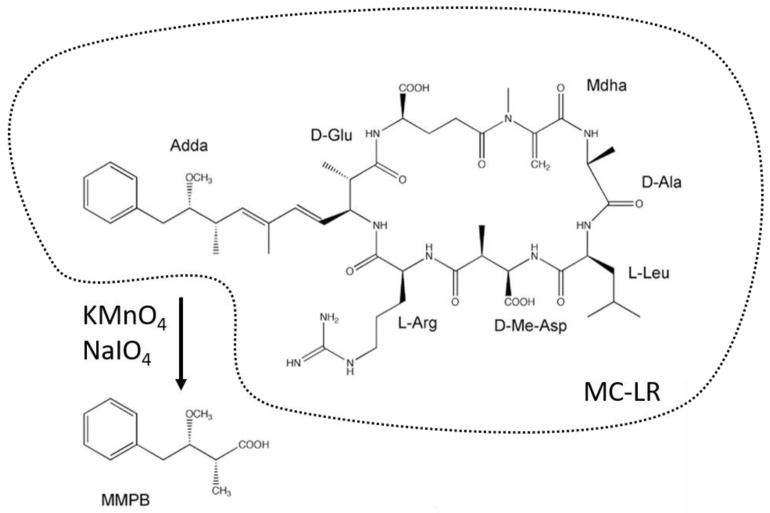
Structure of microcystin-LR (MC-LR) with its specific Adda moiety subject to Lemieux oxidation resulting in MMPB production (modified after Suchy and Berry [61]).

**Figure 3:**
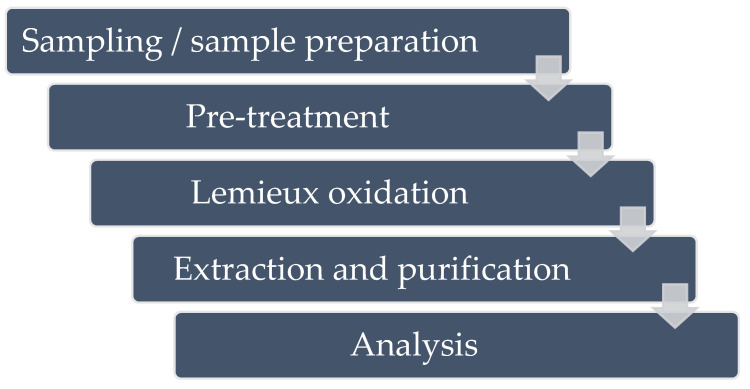
The different stages of MMPB method. These steps have been the subject of optimization work described in the following sections.

Since the 1990s and the development of the first ELISA methods for the analysis of MCs, the need to have a specific antibody for each MC variant caused the analysis to be restricted to a small number of MCs or even only to MC-LR [59,60]. The methodological challenge was thus to develop a method that would be independent of the structural diversity of MC variants. The existence of a unique part common to all MC variants and nodularins, Adda moiety, was the basis of the strategy first developed by Sano et al. [61] for use on cyanobacteria samples (Figure 1). It consists of the oxidation of the Adda part of MCs with potassium permanganate (KMnO_4_) and either sodium-iodate (NaIO_3_) or sodium-periodate (NaIO_4_) to form the corresponding carboxylic acid derivative: the 2-methyl-3-methoxy-4-phenylbutyric acid (MMPB; Figure 2). The oxidation product (MMPB) was analyzed by GC coupled to a flame-ionization detector (FID) or by HPLC coupled to a spectro-fluorimeter. Concerns related to MC accumulation in animal tissues, in particular in aquatic species potentially exposed to cyanobacteria, have led to the identification of a protein-bound fraction of MCs in these biological matrices [16]. The use of the term “bound” toxin usually remains ambivalent as it can sometimes be applied to matrices such as sediments in which MCs are adsorbed on inorganic or organic particles, but also to MCs covalently bound to proteins, in particular PP1 and PP2, in animal matrices [62,63]. However, these covalently-bound forms are not extractable as easily as free (i.e., not protein-bound) MCs in water, in bloom, or in the tissues of an organism. The use of the MMPB method (also called Lemieux oxidation) allows for the extraction of the total (i.e., protein-bound plus free) fraction of MCs. This method was first applied to mussels (*Mytilus edulis*), salmon *(Salmo salar*) (liver tissue), and Dungeness crab larvae (*Cancer magister*) [58,64]. There is a strong disparity in the MMPB method protocols used, as shown in Table 1. Many different matrices were analyzed with a high proportion of matrices of aquatic origin. Other studies have used the MMPB method following animal mortalities in an effort to determine the cause of mortality [57,65,66]. Many steps of the protocol used can possibly influence the final performance of the method, beginning with the pre-treatment of the matrix (Figure 3).

### 2.1. Pre-Treatment of Matrices

In terms of pre-treatment, two approaches can be distinguished depending on the desired purpose: for tissues, enzymatic or chemical treatments are used to begin the digestion of the tissue and facilitate the oxidation of MCs, whereas, for cyanobacterial matrices, the objective will be to break the cells to release the toxins before filtering or pre-concentrating the sample. Concerning the enzymatic/chemical treatments, the first option consists of enzymatic digestion with trypsin, the objective of which is to affect the protein integrity of the matrix in order to facilitate the subsequent oxidation. The duration of the trypsin treatment can vary; early studies involving a trypsinization pre-treatment prior to Lemieux oxidation on freeze-dried *Lymnaea stagnalis* tissues and serum suggested the addition of trypsin at a concentration of 0.5 mg/mL in a phosphate buffer for 2 h at 37 °C [28,49]. Subsequently, a study on juvenile trout (*Oncorhynchus mykiss*) force-fed with MC-LR compared two trypsin contact times (2 and 18 h) and concluded that an 18-h trypsinization time yielded a quantity of MMPB around 20% higher than 2 h [29]. The other option is to use freeze/thaw cycles on the matrix in order to break the remaining cells and liberate the toxins before the addition of NaOH for tissue digestion (50 mM for 2 h; Roy-Lachapelle [62]). For the cyanobacterial matrix, an enzymatic or chemical pre-treatment is not useful; freeze/thaw steps or sonication can be used to release the toxins from the cells [63]. Other protocols involve preliminary steps to prepare the water or cyanobacterial samples, such as filtration (0.22 µM nylon filters) or pre-concentration by SPE [63,64].

### 2.2. Lemieux Oxidation Step

The oxidation step, the key step in the extraction of the total forms of MCs, is the one that has been optimized the most. Although the reagents used in the different studies are the same (KMnO_4_ and NaIO_3_ or NaIO_4_), the yield of the oxidation differs according to several criteria, among which are the concentration, pH, the volume of reagents, and the time of oxidation. Several studies have compared these different criteria in order to identify what could improve the yields obtained following oxidation, taking into account that this yield varies according to the matrix (water, cyanobacteria blooms, sediments, or animal matrices such as tissues or biological fluids).

#### 2.2.1. Cyanobacterial Matrices

Regarding Lemieux oxidation applied to water or cyanobacteria cells, the oxidation was originally performed by Sano et al. [65] for 4 h at 0.024 M KMnO_4_ and 0.02 M NaIO_4_. Later on, other concentrations were adopted: 0.05 M KMnO_4_/NaIO_4_ [66], 0.35 M KMnO_4_/0.4 M NaIO_4_ [64], or 0.5 M KMnO_4_/0.015 M NaIO_4_ [63,66], with oxidation times of 1 h [64,66] or 30 min [63]. Two studies were interested in the impact of varying the oxidant concentration on the yield of Lemieux oxidation. Wu et al. [67] varied the KMnO_4_ concentration from 6.33 to 63.29 mM in saturated NaIO_4_ solution (200 mM) and observed that the yield of MMPB increased by increasing the KMnO_4_ concentration, with the best yields obtained for the initial KMnO_4_ concentrations of 50 mM. These observations are supported by Roy-Lachapelle et al. [66], who tested KMnO_4_ and NaIO_4_ concentrations between 10 and 100 mM, with an optimum concentration of 50 mM. Both studies reported that a concentration of oxidants above 50 mM could cause the degradation of the compounds and significant signal variability. Regarding the pH, both studies have concluded, after testing different pH values from 2 to 10.5, that MMPB yields are better with a low alkaline pH value (pH~9) [66,67]. Concerning temperature, Wu et al. [67] concluded that the temperature did not influence the reaction, which can be held at room temperature after comparing temperatures between 0 and 35 °C at pH 9 and with oxidant concentrations of KMnO_4_ (50 mM) and NaIO_4_ (200 mM). Finally, the reaction time has also been optimized. Reaction times between 0.5 and 5 h were compared for oxidant concentrations between 10 and 100 mM at room temperature and alkaline pH. While after 3 h, a degradation of the Lemieux oxidation product was observed, between 1 and 3 h no significant difference was observed, and the shortest reaction time (1 h) was chosen [66]. The optimization of these parameters resulted in average yields of 86.7% (50 mM KMnO_4_ in saturated 200 mM NaIO_4_ solution at ambient temperature, and at pH~9, with a time reaction between 1 and 4 h) [67] and 103% (50 mM KMnO_4_, NaIO_4_ at ambient temperature, at pH~9, with a time reaction between 1 and 3 h) [66]. The optimized methods have shown higher yields than previous studies, with average yields of 65% (0.35 M KMnO_4_ in saturated 0.4 M NaIO_4_ solution at ambient temperature at pH~9 with a time reaction of 1 h) [64] but close to those obtained by Sano et al. [65] (84–98% yield with 24 mM KMnO_4_ and 20 mM NaIO_4_ at room temperature for 4 h).

#### 2.2.2. Sediment Matrices

For sediments, three studies have focused on improving the oxidation conditions. First, Wang et al. [68] reported that the best oxidation conditions were 0.025 M of KMnO_4_ and 0.093 M of NaIO_4_ for one hour, which yielded 35% of the MMPB. Wu et al. [69] tested a range of KMNO_4_ between 12.5 and 100 mM and concluded that the optimal concentration was 50 mM or more of KMNO_4_. Another study by Bolotaolo et al. [70] demonstrated optimal yield conditions with a reaction time of 3 h with a 0.1 M saturated solution of both KMnO_4_ and NaIO_4_, giving an 89% oxidation yield, which is much better than those obtained earlier (33 to 45%) [68,69].

#### 2.2.3. Animal Matrices

The oxidation of animal matrices contaminated with MCs is a more complex task, as shown by the lower and more variable yields (from 6 to 114% yield, Table 1) and the very different concentrations of oxidants according to the studies (KMnO_4_: from 334 µM to 0.1 M [28,71]; NaIO_4_: from 1.4 mM to 0.1 M [28,60]. Matrix effects can strongly influence the oxidation and extraction efficiencies, and it would be difficult to propose a harmonized protocol suitable for all animal matrices. However, a few protocols have been applied to very different matrices and could therefore serve as a good reference protocol for the analysis of the total forms in animal samples. For example, the protocol proposed by Ott and Carmichael [72] has been applied to fresh human sera, fresh human liver, fresh human blood, and lyophilized terrapin tissue (*Emys orbicularis* and *Mauremys leprosa*) [73,74,75,76]. In this protocol, 1 g of fresh rat or chicken liver was oxidized with 0.02 M of oxidants for 3 h at pH 9. Then in the 2010s, two other protocols seemed to be reused in several studies. The first one is the Neffling and Lance protocol [28,49] on freeze-dried gastropods (*L. stagnalis*) using 0.1 M of both oxidation reagents, which was reused by Cadel-Six et al. [29] on rainbow trout (*Oncorhynchus mykiss*) (lyophilized muscles, liver, intestines, and gills), by Greer et al. [77] on tilapia fishes (*Oreochromis niloticus*) (lyophilized muscles, liver, and eggs), by Brown et al. [78] on a lyophilized sample of coastal bottlenose dolphins (*Tursiops truncatus*), by Foss et al. [59] on lyophilized samples of mallard ducks (*Anas platyrhynchos*), by Mohamed et al. [5] on fresh samples of Nile tilapia (*Oreochromis niloticus*) fish muscles, and by Bolotaolo et al. [70] on lyophilized clams, with varying degrees of extraction efficiency (from 5 to 55%). The second protocol that stands out was proposed by Roy-Lachapelle et al. [66], using 0.05 M of the two oxidant reagents. Although this protocol has not been applied to as many matrices as the previous one, it has been developed on river water and then used on frozen homogenized fish tissues (*Osmerus mordax*; *Mugil cephalus*; *Salvelinus fontinalis*; *Perca flavescens*; *Sander vitreus*; *Ameiurus nebulosus*; *Coregonus clupeaformis*) [79] and algal dietary supplements [80] with satisfactory extraction yields (91%, 54–72%, and 67–74%, respectively). There seems to be disagreement on the concentration of oxidants (0.1 M versus 0.05 M for both reagents for Neffling and Lance’s and Roy’s protocols, respectively) to be used, which depends on both the matrix and the number of toxins to be oxidized. The reaction time for cyanobacterial matrices has to be under 3 h, but Neffling and Lance recommend a time reaction of 3 h in animal matrices against 2 h for Roy-Lachapelle. Both protocols indicate that an alkaline pH between 8 and 9 allows for better results.

### 2.3. Extraction and Purification of the MMPB Product

Purification is another essential part of the MMPB method, allowing the samples to be both cleaned from impurities coming from the matrix and to also be concentrated. Indeed, the use of a large number of salts (KMNO_4_, NaIO_4_, Na_2_HPO_4_, or NaH_2_PO_4_) during this method can negatively affect the performance of the analysis [79]. Furthermore, the volume of the extract (which can be more than 20 mL depending on the protocol) may necessitate a sample concentration. Two different purification methods are mainly used: liquid–liquid extraction (LLE) and solid-phase extraction (SPE). Although the majority of the studies reviewed here use SPE, LLE has been tested on various matrices such as water samples [65,66] but also animal matrices (Patagonian pejerrey (*Odontesthes hatcheri*); tilipa fish; salmon Liver and Dungeness Crab Larvae; saltwater mussels (*Mytilus edulis*)) [5,60,71,81]. Several organic solvents can be used during LLE, notably diethyl ether [60,71,81] and n-hexane [5], but ethyl acetate seems to be the most optimal [65,66]. Indeed, a study has investigated different solvents according to their non-miscibility with water and their influence on the MMPB yield. Ethyl acetate was selected, and the yield was further improved by the addition of a solution of saturated NaCl in the aqueous phase allowing for a reduction in the solubility of the neutral compounds and consequently improving the extraction (salting-out effect) [66]. SPE, the main method for the purification of extracts after the oxidation phase, allows, similar to LLE, cleaning but also the concentration of the samples (with a lower elution volume than the loading one). Among the protocols gathered in Table 1, 16 different types of SPE cartridges were identified. These differences concern the sorbent used (for example, C18, Styrene–divinylbenzene (SDB)), its quantity (from 30 to 500 mg), or the volume of the cartridge. Each type of sorbent used in the cartridge phase has its own properties; the wash and elution parameters are generally optimized and may differ between different SPE protocols. Some studies compared SPE cartridges based on their recovery for a given matrix. Ott and Carmichael [72], for example, compared three SPE cartridges (3M C8-SD^®^, 3M SDB-XC^®^, and Waters Oasis HLB^®^) on chicken and rat liver matrices. Blank liver samples were extracted with each cartridge with the following procedure: conditioning with 1 mL of methanol (MeOH), 1 mL of double distilled water, loading the sample, washing with 0.5 mL of MeOH, and elution with 1 mL of MeOH. The highest signal strength of the Oasis extract indicated that the cartridge did not effectively clean the sample and was subsequently dismissed. To decide between the two remaining cartridges, the MMPB recovery rate was tested, and the SDB-XC cartridge showed a higher recovery (82.2%) compared to the C8 (62.5%) SPE cartridge. Cadel-Six et al. [29] compared the use of two other cartridges, Oasis HLB^®^ (200 mg) from Waters and Agilent SampliQ OPT^®^ (150 mg, Interchim, Montlucon, France) on MMPB standard solutions by varying the elution conditions (MeOH 80%, MeOH 100%, and MeOH 80%, with 0.05% acetic acid (AcOH), 100% AcOH). Whatever the elution conditions tested, the recovery rates obtained were better with the SampliQ OPT^®^ cartridges than with the Oasis HLB^®^. The highest yields among the SampliQ OPT^®^ cartridge tests were obtained by eluting with 80% MeOH (average recovery rate: 80.9%) or with 100% MeOH (average recovery rate: 81.6%). The addition of 0.05% AcOH to 80% MeOH did not increase the recovery rate (average of 78.5%). These conditions were then applied to rainbow trout matrices spiked with 100 ng of MC-LR (equivalent (eq.) to 20.9 ng of MMPB) and 50 ng of MC-LR (eq. to 10.5 ng of MMPB) overnight at 4 °C. The SPE extraction efficiency was 25% for the intestines, 19% for the muscles, 28% for the liver, and 18% for the gills. An important fraction of MMPB is either not extracted, degraded, or not retained by the cartridge used. Another possibility is the application of an on-line SPE method for the purification of the sample after Lemieux oxidation. Unlike the classical SPE methods for cartridges (off-line SPE), the on-line-SPE is directly coupled with HPLC, saving time on the analysis compared to the off-line methods. Munoz et al. [64] compared three on-line SPE columns (C18^®^, BetaBasic-18^®^, and Hyper-Sep Retain PEP^®^) on environmental surface water samples. Some peak-tailing was observed with the Hyper-Sep^®^, while the C18^®^ could not efficiently withstand a large number of consecutive injections. Due to the difference in pH between the samples (pH around 2 after the oxidation step) and the solution used during the washing step, 0.5% *v*/*v* formic acid was added to the mobile phases of the on-line SPE. According to the authors, this washing step is the most critical as it allows the desalting and removal of interfering matrix components. Therefore, the optimized method corresponds to a sample loading of 5 mL and a wash with 3 mL, both at a flow rate of 2500 µL/min. By using this on-line SPE on water samples, MMPB was analyzed in just 8 min with a limit of quantification (LOQ) of 0.5 ng/L. Another derivative of the SPE method that could be applied to total MCs is solid phase micro-extraction (SPME). This method uses a fused polymer-based fiber as an adsorption medium for the analyte [82]. SPME was first developed with fish tissues (*Goodea* sp.; *Oncorhynchus nerka*; *Perca flavescens*) spiked with MCs and then applied to environmental fish samples [61]. Due to the detection mode used (GC-MS), the purified extracts had to be derivatized to form meMMPB. Three fiber types (Supelco, Inc., Belfort, PA, USA) were compared: polydimethylsiloxane/divinylbenzene (PDMS-DVB), divinylbenzene/Carboxen™/polydimethylsiloxane (DVB-CAR-PDMS) and polyacrylamide (PA). Among these three types of fiber, the best linearity between the peak area and concentration of MMPB was obtained with PDMS-DVB, and an LOQ of 0.14 µg/g was calculated. Following purification, it is possible to add an intermediate step before analysis and to concentrate the extracts by evaporating under nitrogen [29,70] or argon flow [28] at different temperatures from 25 to 40 °C [29,49,70,83].

### 2.4. Analysis of the MMPB Product

The majority of the studies concerning the MMPB method use mass spectrometry (MS) as a detector coupled with either liquid or gas chromatography as the method of analysis. Regarding the conditions under which the chromatography is performed, whether liquid or gas, different chromatographic columns are used in the studies that are listed in Table 1. Nevertheless, the parameters used during the MS analysis can be listed. The positive ionization mode of MS is used in many studies [28,29,49,77]. The most commonly searched fragments in this ionization mode, after electron ionization in GC MS, is m/z 176 [60,71,75]. In some studies, the fragment analyzed by GC-MS is not MMPB but methyl-MMPB [76]. In this case, the MMPB is methyl-esterified before the analysis in order to volatilize the MMPB for the analysis. This step is performed by using, for example, 1 mL of hydrochloric MeOH (5%) and silver carbonate followed by a heating step at 70 °C for 1 h [61,70] or by using 12% trifluoroborate in MeOH [84]. In LC-ESI/MS, the parent ion [M + H] + *m*/*z* 209 is monitored along with its three specific fragments at *m*/*z* 91; 131 and 191 [28,49]. However, several studies report better results using the negative ionization mode for MS/MS (207 → 131 transitions) [59,63,64,72,85,86,87]. Some alternatives to the classical LC-MS/MS methods exist, namely laser diode thermal desorption-atmospheric pressure chemical ionization coupled to tandem mass spectrometry (LDTD-APCI-MS/MS) [66,79] and condensed phase membrane introduction mass spectrometry (CP/MIMS) [88]; both methods were applied to aqueous samples with a detection limit of 10 µg/L and 1 mg/L, respectively. Less commonly, other detection systems can be coupled to HPLC for the analysis of the MMPB product generated during Lemieux oxidation, HPLC with photodiode array detection (PDA) [89], HPLC with ultra-violet detection (UV) [63,67,69,90], or HPLC with fluorescence detection (FLD) [68]. Inversely, the use of the Adda ELISA kit on oxidized drinking water samples did not enable the detection of the MMPB fragment following the MMPB method [63].

### 2.5. Limitations and Difficulties Faced during the MMPB Method

#### 2.5.1. Sample Turnaround Time

One of the first obstacles to the MMPB method concerns the processing time of a sample. Steps such as an enzymatic pre-treatment with trypsin can range from 2 to 18 h. Moreover, the work by Cadel-Six et al. [29] showed that the most efficient trypsin pre-treatment lasted 18 h. This step is followed by the oxidation step, which can last several hours (1 to 3 h). Then, depending on the strategy chosen, other time-consuming steps are to be undertaken: filtration (sometimes with important volumes), LLE or SPE purification, and the concentration of the samples. The realization of the oxidation proves to be both time-consuming and fastidious due to the number of steps to realize it, which increase the sample turnaround time and can limit the simultaneous processing of a large number of samples.

#### 2.5.2. Variability of the Overall Conversion Process

The review of the literature on the MMPB method has shown that the conversion yields strongly differ among matrices (i.e., cyanobacterial matrices, sediments, and animal matrices). The yields for cyanobacterial matrices and sediments are around 80%, and those for animal matrices range from 4% for dog hair to 114% for a golden retriever (*Canis lupus familiaris*) vomit sample [86]. Even for the same protocol, significant variability can be observed depending on the matrices and for the same animal species depending on the organs, probably in relation to the levels of lipids and proteins in the tissues and the associated efficiency of MMPB extraction. Three times higher yields were thus observed on trout liver compared to trout muscle [29], while in the study by Foss et al. [86], the oxidation yield for dog hair was only 4%, while this yield was 114% for dog vomit samples. When considering the performance of the different steps taken one by one, a strong disparity can be observed. The study by Cadel-Six et al. [29] thus proposes the yield of the method for the intestinal tissue of rainbow trout but also the yield of the various stages of the method: 25% for the oxidation step, 93% for filtration, and 27% for the SPE + evaporation step. Globally, the MMPB protocol provides an overall yield of 6%. Switching to another type of tissue, gills provide better yields in terms of oxidation (67%) but much lower yields for SPE and evaporation (18%), giving a final overall yield of the method of 12% (it is to be specified that for the gill matrix, the filtration yields have not been specified). Even with an optimized protocol, switching from one animal matrix to another can have an impact on the yield and shows how important the matrix effect can be. These results can be compared to the study by Bolotaolo et al. [70], also providing the yields of their step-by-step MMPB method on two matrices: sediments and clams at two spike levels of MC-LR (100 and 500 µg/L). The protocol begins with a lipid extraction step with average yields of 96% for sediments and clams. The main losses estimated in this method occurred during the conversion step of MC-LR to MMPB, with average conversion rates of 75% for sediments and 64% for clams. It should be noted that this study distinguishes between conversion and oxidation yields without making this distinction explicit, which could lead to confusion when comparing the yields of this method to another. Much higher yields than the method used by Cadel-Six et al. [29] were observed in the method used by Bolotaolo et al. [70] during the oxidation and SPE steps, with average yields for sediments of 91% and 91%, respectively, and for clams of 91% and 91%, respectively. Thus, the step that seems to generate the most loss is oxidation, with yields ranging from 25% to 60% for intestinal tissue and gills, respectively, in Cadel-Six et al. [29], and a yield of about 91% for sediments and clams in Bolotaolo et al. [70].

#### 2.5.3. Matrix Effects

One reason that can be advanced to explain this loss of MMPB could be the matrix effect. In their study, Munoz et al. [64] distinguished between instrumental matrix effects and matrix effects that can occur during sample preparation. Instrumental matrix effects are often caused by co-eluting matrix components that can lead to ion suppression or enhancement phenomena at the ionization source [91], while sample preparation matrix effects can occur when an incomplete extraction recovery is achieved from the test samples [64,92]. Regarding the matrix effects related to water samples when using the MMPB method, Munoz et al. [64] determined instrumental matrix effects ranging from −59% to −75% and minimal matrix effects during the oxidation step. In addition, the analysis of environmental water samples by LDTD-APCI-MS/MS allowed for oxidation yields of 103% with a recovery signal of MMPB at 91%, showing the absence of a significant instrumental matrix effect during the analysis [66]. In another method developed by Roy-Lachapelle et al. (2015) using LDTD-APCI-HRMS on fish tissues, quite low instrumental matrix effects were observed (between 7 and 10%). Of the few studies analyzing the total forms of MCs in sediments, little information is reported on the matrix effects. The only information available indicates that there is “an adverse impact of the sediment matrix on Lemieux oxidation yields” without quantifying this impact [69]. In the animal matrix studies, instrumental matrix effects leading to either signal gain or loss were both observed. The study by Ott and Carmichael [72] showed a signal suppression of 41.8% in rat and chicken liver, and Neffling et al. [49] observed a signal loss in the serum and tissue of aquatic snail samples spiked at the final step of sample preparation (after oxidation and extraction) ranging from 63 to 84%. On the contrary, other studies report a signal enhancement effect on rainbow trout samples extracted and spiked just before injection, where the recovered signal was 122% for the intestines and 142% for the muscles [29]. Similarly, a study conducted on *Dreissena polymorpha* tissues estimated the signal enhancement to be 300% [93]. Although two types of matrix effects (instrumental and sample preparation) have been distinguished, most of the data collected on the MMPB method’s protocols seem to focus only on instrumental matrix effects.

#### 2.5.4. Quantification Strategy (Internal vs. External Calibration)

Different quantification strategies have been set up for the analysis of total MCs after Lemieux oxidation, attempting to compensate for the matrix effects that can be more or less impactful depending on the type of matrix or the analytical instrument used. Anaraki et al. [87] already listed some of the studies on the MMPB method, including the quantification strategy and the use of an internal standard. This inventory was extended with the objective of gathering studies using the same strategy (or a similar one). The first quantification strategy adopted in MMPB method studies corresponds to an external calibration. The quantification of the MMPB in samples can be performed using an MMPB calibration curve made of an MMPB standard [5,29,67]. Another possibility is using an MMPB calibration curve generated by the oxidation of an MC-LR standard [81]. The disadvantage of these first two strategies is that they do not take into account the presence of the matrix in the samples and, therefore, the matrix effects. Moreover, generating an MMPB standard curve after the oxidation of the different MC-LR concentrations adds an additional bias related to the oxidation efficiency. More common in the studies reviewed is the use of an external matrix-matched calibration. Greer et al. [77], for example, chose to spike the matrix with MMPB after the pH adjustment step, while other studies preferred to use an in situ (here in the sense of calibration curve prepared in the matrix) matrix-matched calibration curve of MC-LR-fortified samples that subsequently went through the oxidation process to generate an MMPB matrix-matched calibration curve [60,71,86,93,94]. Finally, one study used the standard addition approach, consisting of spiking the analyzed samples at three MC-LR levels prior to oxidation, to quantify the MMPB in the liver samples [78]. Another strategy is to use an internal calibration via an internal standard (IS). For MMPB analysis, two IS can be used, 4-phenylbutyric acid (4-PB) [66,79,90] and isotopically-labeled MMPB (d3-MMPB) [84,88]. The first factor of choice between the two IS is the availability of the product; indeed, while 4-PB is easily available, d3-MMPB is more expensive and more difficult to obtain. On the water matrix, Munoz et al. [64] provided a second factor of choice by comparing the deviation between surface water samples and reference HPLC-water samples, both oxidized and spiked with either 4-PB or d3-MMPB. They observed a deviation of 14% for 4-PB and of around 5% for d3-MMPB, concluding that these IS allow a sufficient correction of the matrix effect whatever the IS used. A different quantification comparison was performed by Anaraki et al. [87] on a fish matrix between a calibration curve using an MMPB standard (not generated from MCs) and an in situ MMPB generated matrix-matched standard curve, both using d3-MMPB as IS. After 2 h of oxidation, the recovery of d3-MMPB was much higher when calculated using the standard curve prepared with the MMPB standard (78.9%) rather than with the generated MMPB (30%), resulting in a lower relative response (MMPB/d3-MMPB) than with a calibration curve using MMPB standard. Thus, according to the tests performed in this study, the MMPB calibration curve generated in situ was three times lower than the one using the MMPB standard. The consequence is an underestimation of the amount of MMPB present in a sample when the curve that is generated in situ is used. It is therefore strongly recommended to use an IS to avoid any bias in the results of the analysis. Some studies also use a dual approach with external and internal quantification [63,70,72]. In Foss and Aubel [63], for example, the combination of a standard curve of oxidized MC-LR and 4-PB as IS was used for quantification.

**Table 1:** A comparison of the various conditions of the MMPB method protocols in scientific publications (modified after Anaraki et al. [87]). (n.s.: not specified; dw: dry weight).

References	Matrix		Quantity of Matrices	Pre-Treatment Step	Oxidation Length (Hour)	[KMnO_4_]	[NaIO_3_] or [NaIO_4_]	pH	End of Reaction	Purification Method	Detection Limits	Instrumental Analysis	Yield
Sano et al. [65]	MC fraction from waterblooms (*Microcystis viridis*)		0.2–1 mg (dw)	-	4	0.024 M	0.02 M	n.s.	0.04 mL of 20% NaHSO₃ + 0.01 mL of 1OM H_2_SO_4_	5 mL of ethyl acetate	pmol	GC-FID/HPLC	84–98%
Williams et al. [60]	Salmon Liver (*Salmo salar*)		220–1366 mg	-	overnight	36.1 µM	1.3852 mM	9 (120.8 µM of K_2_CO_3_)	NaHSO₃ + 10% H_2_SO_4_ (pH~2)	Diethyl ether	15 µg/g	GC/MS	85–95%
	Dungeness Crab Larvae (*Cancer magister*)		5.2–5.4 g										n.s.
Williams et al. [71]	Saltwater mussels, (*Mytilus edulis*)		5.5–8.3 g	-	n.s.	334.2 µM	12.3 mM	9	NaHSO₃ + 10% H_2_SO_4_ (pH ≈ 1–2	Diethyl ether	15 µg/g	GC/MS	n.s.
Pires et al. [93]	Zebra mussels (*Dreissena polymorpha*)		Pooled mussel material	-	overnight	0.1 M	0.4 M	9	H_2_SO_4_ (1 M), pH < 3	C18 (Supelco)	n.s.	LC/MS	n.s.
Ott and Carmichael [72]	Rat or chicken livers (species n.s.)		0.5–1 g	-	3	0.02 M	0.02 M	9 (KHCO_3_)	0.5–1.5 g of NaHSO₃ + 10% H_2_SO_4_ (pH ≈ 2)	3 M Empore SDB-XC 7 mm/3 mL (Fischer Scientific, Hampton, NH, USA)	5 µg/g	LC/MS	33.5%
Soares et al. [74]	Human sera		0.4–1 mL	-	3	0.02 M	0.02 M	9 (KHCO_3_)	0.5–1.5 g of NaHSO₃ + 10% H_2_SO_4_ (pH ≈ 2)	3 M Empore SDB-XC 7 mm/3 mL (Fischer Scientific)	n.s	LC/MS	n.s.
Yuan et al. [75]	Human Liver/sera		n.s.	-	3	0.02 M	0.02 M	9 (KHCO_3_)	0.5–1.5 g of NaHSO₃ + 10% H_2_SO_4_ (pH ≈ 2)	3 M Empore SDB-XC 7 mm/3 mL (Fischer Scientific)	n.s.	GC/MS	n.s.
Hilborn et al. [73]	Human blood		n.s.	-	3	0.02 M	0.02 M	9 (KHCO_3_)	0.5–1.5 g of NaHSO₃ + 10% H_2_SO_4_ (pH ≈ 2)	3M Empore SDB-XC 7 mm/3 mL (Fischer Scientific)	n.s	LC/MS	n.s.
Nasri et al. [76]	Terrapin (*E. orbicularis*; *M. leprosa*)		100 mg (dw)	-	3	0.02 M	0.02 M	9 (KHCO_3_)	0.5–1.5 g of NaHSO₃ + 10% H_2_SO_4_ (pH ≈ 2)	Conversion of MMPB to methyl ester using 12% trifluoroborate in MeOH	n.s.	GC/MS	n.s.
Wu et al. [90]	Aqueous solution spiked with MC-LR		-	-	hours	50 mg/L	20 mg/L	1.6	NaHSO₃	-	n.s.	HPLC/DAD	~90%
Wu et al. [67]	Cyanobacterial samples (*Microcystis* spp.)		10; 20; 40; 60; 80; 100 mg (dw)	-	1–4	≥0.05 M	0.2 M	9 (KHCO_3_)	Saturated NaHSO₃ sol.	-	n.s	LC/DAD	86.7%
Lance et al. [28], Neffling et al. [49]	Tissues of snails (*Lymnaea stagnalis*)		10 mg (freeze-dried)	Trypsin in Sörensen’s phosphate buffer (0.5 mg/mL, pH 7.5, 37 °C)	3	0.1 M	0.1 M	9	40% NaHSO₃ + 10% H_2_SO_4_	Oasis HLB 30 mg	n.s.	LC/MS	16–37%
Suchy and Berry [61]	Rainbow Trout (*Oncorhynchus mykiss*) liver slurry (7 g in 5 mL of deionized water)		1 mL of liver slurry	-	3	0.0035 M	0.09 M	9	0.5–1.5 g of NaHSO₃ + 10% H_2_SO_4_ (pH ≈ 2)	Solid phase microextraction (PDMS-DVB fibre)	0.04 µg/g	GC/MS	n.s.
Wu et al. [69]	Lake sediment		2 g (freeze dried)	-	1–4	0.05 M	0.1 M	9	Saturated NaHSO₃ sol.	Sep-pak C18 500 mg (Waters)	n.s.	HPLC/DAD	33-45%
Bieczynski et al. [81]	Supernatants of liver and intestines from fish (*Odontesthes hatcheri*)		1 g	-	overnight	0.002 M	0.007 M	9	20% NaHSO₃ + drops of 10% H_2_SO_4_	Diethyl ether	0.2 ng	GC/MS	67.25 ± 26%
Cadel-Six et al. [29]	Rainbow trout (*Oncorhynchus mykiss*)	Liver Intestines Gill Filet	60 mg 100 mg 100 mg 100 mg (dw)	Trypsin in Sörensen’s phosphate buffer (0.5 mg/mL, pH 7.5, 37 °C)	4	0.1 M	0.1 M	9	40% NaHSO₃ + 10% H_2_SO_4_ (pH ≈ 1.5)	SampliQ OPT 150 mg (Agilent)	2.59 ng/g 4.76 ng/g 2.86 ng/g 2.88 ng/g	LC/MS	17% 6% 12% 5%
Roy-Lachapelle et al. [66]	River water		n.s.	-	1	0.05 M	0.05 M	9 (K_2_CO_3_)	40% NaHSO₃ + 10% H_2_SO_4_ (pH ≈ 2)	Ethyl acetate	0.2 µg/L	LDTD/APCI/MS	91%
Roy-Lachapelle et al. [79]	Frozen homogenized fish tissue ((*Osmerus mordax*); (*Mugil cephalus*); (*Salvelinus fontinalis*); (*Perca flavescens*); (*Sander vitreus*); (*Ameiurus nebulosus*); (*Coregonus clupeaformis*))		1 g	3 freeze-thaw lysis cycles + 2 h in NaOH (50 mM)	2	0.05 M	0.05 M	9 (HCl, 50 mM)	Saturated NaHSO₃ sol. + 10% H_2_SO_4_ (pH ≈ 2)	Filter the supernatants with 0.2 µM nylon filter (Whatman) + Strata SDB-L 500 mg (Phenomenex)	2.7 ng/g	LDTD/APCI/HRMS	54–72%
Foss and Aubel [63]	Raw and trated water from Ohio water sources		n.s.	3 freeze-thaw cycles + 10/100x concentration (SPE)	0.5	0.015 M	0.015 M	n.s.	40% NaHSO₃ + phosphate buffer to raise pH < 5	Strata X 100 mg (Phenomenex)	0.05 µg/L	LC/MS	n.s.
Wang et al. [68]	Water Sediment		0.1 mL 1 g	-	1	4 g/L	20 mg/L	n.s.	40% NaHSO₃ (50 µL: water/0.5 mL: sediment)	Sep-Pak cartridge (500 mg, 6 cm^3^)	125 ng/L 100 ng/g	HPLC/FLD	98–109%
Greer et al. [77]	Tilapia (*O. niloticus*) filets, livers and eggs		50 mg (dw)	-	2	0.1 M	0.1 M	n.s.	40% NaHSO₃ + 10% H_2_SO_4_ (pH ≈ 2)	Oasis PRiME 60 mg (Waters)	n.s.	UPLC/MS	55%
Munoz et al. [64]	Surface Water		10 mL	3 freeze-thaw cycles + filtration (0.22 µM nylon filters)	1	0.35 M	0.4 M	9 (K_2_CO_3_, 1 M)	NaHSO₃ (4 M)	Filtration (0.22 µM nylon filters) + on-line SPE	0.5 ng/L	LC/MS	65%
Roy-Lachapelle et al. [80]	Algary dietary supplements		0.3 g	-	2	0.05 M	0.05 M	9 (K_2_CO_3_)	Saturated NaHSO₃ sol. + 10% H_2_SO_4_ (pH ≈ 2)	Filtration (0.2 µM nylon filter, Whatman) + Strata SDB-L 500 mg (Phenomenex)	0.2 µg/g	LDTD/APCI/HRMS	67–74%
Vudathala et al. [94]	Liver and plasma from channel catfish (*Ictalurus punctatus*)		<0.5 g/0.08–0.18 g	-	3	0.002 M	0.002 M	9 (Na_2_CO_3_, 1 M)	NaHSO₃ + 0.5 mL of concentrated H_2_SO_4_ (pH ≤ 2)	Filtration (0.2 µM nylon filter, Whatman) + Oasis HLB 60 mg (Waters)	5.2–6.2 ng/g	LC/MS	31%/21%
Brown et al. [78]	Bottlenose dolphins liver (*T. truncatus*)		n.s.	-	2.5	0.1 M	0.1 M	n.s. (K_2_CO_3_, 0.2 M)	40% NaHSO₃	Strata-X 200 mg (Phenomenex)	1.3 µg/g (dw)	LC/MS	39%
Duncan et al. [88]	Aqueous samples/Algal reference material suspensions		n.s./25 mg (dw) in 200 mL of water	-	1.5	0.025 M	0.009 M	9 (KHCO_3_, 0.5 M)	40% NaHSO₃ + HCl (6 M, pH ≈ 3)	Semi-permeable hydrophobic membrane (PDMS)	1 µg/L	CP/MIMS	n.s.
Foss et al. [59]	Mallard duck (*Anas platyrhynchos*) liver		100 mg (dw)	-	2	0.25 M	0.25 M	KHCO_3_ (1 M)	40% NaHSO₃ + 50% H_2_SO_4_ (pH < 2)	Ethyl acetate + Strata-X 200 mg (Phenomenex)	n.s.	LC/MS	n.s.
Greer et al. [95]	Porcine tissue		50 mg (dw)	-	2	0.1 M	0.2 M	n.s.	40% NaHSO_3_ + 10% H_2_SO_4_	Oasis PRiME HLB (Waters)	n.s.	UPLC/MS	83%
Foss et al. [86]	Dogs (*Canis lupus familiaris*)	Liver Kidney Hair Blood Bile Urine Vomit	100 mg 100 mg 50 mg (dw) 0.5–1.5 mL 0.2–500 µL 0.2–500 µL 10 mg	-	2	0.1 M	0.1 M	(K_2_CO_3_, 0.2 M)	40% NaHSO₃	Strata-X 200 mg (Phenomenex) + filtration (PVDF; 0.2 µM; Sigma)	4 ng/g 4 ng/g 20 ng/g 0.2 ng/mL 50 ng/mL 0.2 ng/mL n.s.	LC/MS	21% 12% 4% 28% 72% 42% 114%
Mohamed et al. [5]	Nile tilapia (*Oreochromis niloticus*) filet		n.s.	As described in Neffling et al. [49]						n-hexane	n.s.	LC/DAD	n.s.
Anaraki et al. [87]	Rainbow Trout (*O. mykiss*) liver and filet		100 mg (dw)	-	2	0.3 mM	0.02 M	8.5	NaHSO₃ + 10% H_2_SO_4_ (pH = 3)	Oasis HLB 400 mg (Waters) + Pall GHP filters	7.28 ng/g	LC/MS	30%
Bolotaolo et al. [70]	Sediments/clams		1/0.1 g (dw)	-	3	0.1 M	0.1 M	9	NaHSO₃ (1–1.5 g) + 10% H_2_SO_4_ (pH = 2)	ENV-Bond Elut 100 mg (Agilent) + esterification	10/15 ng/g (dw)	GC/MS	50%/46%
Lepoutre et al. [83]	Mussels (*A. anantina* and *D. polymorpha*)		Freeze-dried tissues	Trypsin in Sörensen’s phosphate buffer (pH 7.5, 37 °C)	3	0.025 M	0.025 M	9	NaHSO₃ + 10% H_2_SO_4_ (pH < 3)	SPE 60 mg (Waters)	n.s.	LC-MS/MS	32.9–58.8/36.5–57%

## 3. Quantitation of Total MC after Ozonolysis

First described by Harada et al. [96], ozonolysis has historically been the second method for screening total MCs in cyanobacteria. Ozonolysis results can be associated with MMPB method products as ozonolysis produces the same fragment. The reaction of MC oxidation by ozone in a bloom sample from a Japanese lake was completed within 30 min, and the products were analyzed by thermospray-liquid chromatography/mass spectrometry (TSP-LC/MS) or electron ionization-gas chromatography/mass spectrometry (EI-GC/MS). The ozonolysis method has also been applied to sediments [97]. On this matrix, MCs tend to be adsorbed [98], causing the analysis to be more complex than in water samples; a liquid–liquid extraction step (three successive partitions with n-hexane) was added. A methylation step (with 14% boron trifluoride-methanol (BF3-MeOH) at 70 °C for one hour) that made the MMPB volatile for GC-MS analysis was also added. The detection limit of this method was evaluated at the pmol levels with yields of 90.5% for MC-LR and -RR and 91.7% for MC-YR. In the most recent application of ozonolysis on MCs, lake water samples spiked with MCs were used to test a slightly different analytical method [99]. After ozonolysis with an ozone concentration of 2.8 mg/L at a flow rate of 100 mL/min for 1 to 20 min, the excess ozone was removed with a nitrogen stream for 5 min. As in the method of Tsuji et al. [97], LLE steps were used. Unlike the sediment ozonolysis method, methyl esterification was performed with MeOH, pyridine, and methylchloroformate (MCF), while the LLE was performed with chloroform in a saturated sodium chloride solution followed by GC/MS analysis. When compared to the previous method using 14% BF_3_-MeOH at a high temperature for at least an hour, the Zhang et al. [99] method showed yields between 77.7 and 80.2%, depending on the spike level (300 and 100 µg/L, respectively). The LOD of this method was 0.34 µg/L for a shorter reaction time (10 min), at room temperature and under mild conditions. Although this method can be performed at room temperature, Zhang et al. [99] showed that the most optimal conditions for this ozonolysis protocol were a reaction with an ozone concentration of 2.8 mg/L for 10 min at an acidic pH (pH ≤ 3) and with a temperature of 35 °C. To our knowledge, these are the only publications using ozone for an analysis of the total forms of MCs. Subsequently, the use of ozone on water containing MCs was performed in the context of treating micro-pollutants in water by ozonation reaction for remediation purposes [100,101,102,103,104,105]. Although this method yielded good results for blooms (77.7–80.2%) [99] and sediments (between 90.5 and 91.7 depending on the MCs variants) [97], to our knowledge, it has never been applied to more complex matrices, such as animal matrices. As mentioned previously regarding the MMPB method, the transition to such matrices can lead to a significant decrease in the yields of total MCs due to more significant matrix effects. Another point of vigilance concerns the term “total” form. Indeed, this term, depending on the matrix to which it refers, does not necessarily refer to the same type of binding between the MCs and the matrix. In the study by Harada et al. [96], using ozonolysis on cyanobacterial blooms only refers to intra/extracellular MCs, whereas in the other case, dealing with MCs adsorbed to sediment, the binding does not correspond to MCs covalently bound as in animal matrices. While being an interesting and fast method to obtain good recoveries of the total MCs on bloom and sediment matrices, more experience is needed in the application of ozonolysis on animal matrices to better appreciate the performances of this method, which nevertheless requires adapted equipment in the laboratory.

## 4. Quantification of Total MC after Base-Catalyzed Deconjugation

Conjugated forms of MCs are often present in natural samples, sometimes at much higher concentrations than free MCs [59,106,107,108,109]. One of the drawbacks of the analysis of total MCs when using the MMPB method is the loss of information on the MC variants. Indeed, the oxidative cleavage at the Adda fragment only preserves the MMPB fragment, common to all MCs and nodularin. It has been observed in the past that the Michael addition of thiols to MCs was reversible [110,111,112]. Different studies have then focused on the phenomenon of the deconjugation of MCs. The first studies on the subject focused on MCs that are synthetically conjugated in a laboratory [113,114,115] and then on tissues naturally contaminated with cyanotoxins and containing free and bound MCs [59]. Although this deconjugation can occur at neutral pH, studies have shown that deconjugation at basic pH increases the rate and efficiency. Two research teams are interested in deconjugation at basic pH as an alternative to the MMPB method.

### 4.1. Zemskov’s Approach of Based-Catalyzed Deconjugation

This protocol was inspired by a mild method for the cleavage of the thioether bond of an S-ethylcysteine residue contained in a modified serine protease mutant by using O-mesitylenesulfonylhydroxylamine (MSH) [116]. This protocol was adapted to MCs by focusing on a laboratory-conjugated MC model. The model system was prepared by adding a glutathione (GSH) fragment as a model of a protein phosphatase to an MC-LF fragment. The addition of GSH to the Mdha residue of MC was performed smoothly by adding 25 equivalents of K_3_PO_4_ at 40 °C. The model compound thus corresponds to an asymmetric thioether derivative that can be considered to be an N-Me-lanthionine-linked peptide. First, the model compound was subjected to electrophilic activation with excess MSH and potassium carbonate in dimethylformamide/water. However, the need for excess MSH caused the purification of the deconjugated MC fragment to be difficult, and an alternative method of cleaving the thioether bond was sought. After observing a partial cleavage of the thioether bond upon the treatment of the model compound with a base under harsher conditions, as well as the formation of a product whose mass corresponded to the deconjugated MC from the model compound, Zemskov et al. [115] wanted to investigate the possibility of a deconjugation of MC following a basic treatment. The model compound was treated with 20 equivalents of K_3_PO_4_ at 90 °C under microwave irradiation, and the cleavage of the model compound was observed by LC-MS analyses and isolated by HPLC with a yield of 77%, but further NMR analyses showed that the product obtained was a cyclic product. This cyclization is explained by the intramolecular attack of the N-terminus on Mdha. To overcome this, the addition of propargylamine allows for the trapping of the Michael acceptor and avoids the formation of the cyclic product obtained in the previous reaction. To confirm that the developed method could be applied to adducts on natural MCs, an MC-LF/GSH was synthesized and dissolved in a mixture of propargylamine/MeOH (1:1) before the addition of KOH (final concentration = 0.09 M) in order to initiate the cleavage of the MC-LF/GSH. Two diasteromeric forms of propargylamine conjugated to MC-LF were identified in the LC-MS analysis, confirming the deconjugation of MC-LF to GSH.

### 4.2. Miles’ Approach of Based-Catalyzed Deconjugation

#### 4.2.1. Based-Catalyzed Deconjugation of Thiol-Conjugated MCs 

**Figure 4:**
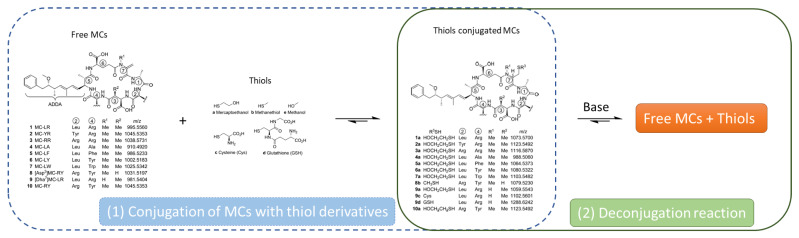
A schematic representation of (1) the mechanism of conjugation of the Mdha^7^/Dha^7^ moiety of MCs with different thiols to create MCs-thiols derivatives as model compound for (2) deconjugation reaction with base (modified after [114]).

Miles et al. [114] studied the deconjugation kinetics of some thiol-conjugated MCs (Figure 4). Different variants of MCs (MC-LR, -YR, -RR, -LA, -LF, -LY, -LW, -RY, [Asp^3^]MC-RY, and [Dha^7^]MC-LR) were conjugated to different nucleophilic compounds, such as mercaptoethanol, methanethiol, MeOH, and cysteine or glutathione. A base-catalyzed conjugation was performed to obtain the different thiol-conjugated MCs. Once synthesized, the stability of these MC-thiol conjugates was studied under basic conditions, e.g., the MC-RY conjugate with mercaptoethanol was deconjugated under weakly basic conditions, and the half-life of the conjugate decreased when the pH increased or when DMSO or iodoacetamide was added. Subsequently, further kinetic studies on eight MC conjugated with mercaptoethanol were performed using smooth deconjugation using a MeOH-carbonate buffer. At pH 10.7, most of the thiol derivatives had half-lives ranging from 30 to 50 h. This shows the possibility of deconjugating conjugated MCs, and further experiments were subsequently performed to accelerate the process. Three possibilities have been put forward to try to speed up MC deconjugation, firstly by increasing the pH in order to increase the rate of deconjugation while minimizing the occurrence of side reactions. The second possibility put forward by Miles et al. [114] is to remove the free thiol formed during the reaction in order to drive the reaction equilibrium towards deconjugation. Finally, the last possibility is in accordance with the authors who converted the free MCs into non-thiol-reactive derivatives in order to avoid the reconjugation phenomena. To do this, one solution is to carry out the reaction in the presence of an excess of non-natural thiols, such as mercaptoethanol. These hypotheses have been considered and were successfully demonstrated on thiol-conjugated MCs [114,115]. However, it has been observed that the use of high pH can lead to artifact formation [114] and that the use of derivatization reagents can lead to the formation of isomers, complicating the subsequent chemical analysis [114,115].

#### 4.2.2. Sulfone/Sulfoxide Mediated Deconjugation 

Another hypothesis considered was that the deconjugation reaction could be improved by first converting the sulfide group of the MC to a more easily-leaving group, such as sulfoxide or sulfone [113]. This strategy allows closeness to a more-known reaction: the base-catalyzed β-sulfone elimination reaction [117,118]. As in the previous study, MCs conjugated to thiol derivatives (e.g., mercaptoethanol) were used as a model compound. These compounds with a dialkyl sulfide linkage were oxidized to either sulfoxide or sulfone, which upon basic treatment removed the thiol adduct to its sulfenate or sulfinate to recover a deconjugated MC upon β-elimination with the α, β-unsaturated carbonyl group intact. Sulfone-mediated deconjugation was performed on the mercaptoethanol conjugate of MC-RY with oxone in a methanolic carbonate buffer. The rapid and complete conversion of this conjugate to its free form MC-RY via the sulfone form was achieved, and after 26 min of reaction, the conjugate form was no longer detachable. On the other hand, after one night, a drop by half in the intensity of the MC-RY peak was observed and attributed to the instability of MC with oxone. The possibilities put forward to remedy this would be either to quench the reaction after the formation of the sulfones or to extract the products by SPE to remove excess oxidant, for example, by adding a sacrificial sulfoxide such as Me_2_SO [119]. In this study, the other hypothesis tested was sulfoxide-mediated deconjugation. H_2_O_2_, which is known to oxidize the Met (methionine) residue of MCs to their sulfoxide, was used to oxidize the methanethiol conjugate of [Asp^3^]MC-RY and the mercaptoethanol conjugate of eight other MC variants. All of these conjugates were oxidized and then separated by SPE from the excess oxidant before being deconjugated in a carbonate buffer at pH 10.7. Between these two mediated deconjugations, the study of the reaction kinetics between sulfones and sulfoxide-mediated deconjugation showed that the sulfones were thus deconjugated twice as fast as their corresponding sulfoxide. Thus, an optimized sulfoxide-mediated deconjugation procedure using Me_2_SO was developed and applied to a mixture of eight MCs conjugated to a mercaptoethanol residue. This experiment was performed as a one-pot reaction in an LC vial, first by adding Me_2_SO and then oxone, and one to two hours later, a carbonate buffer was added to start the deconjugation. The deconjugation process was almost complete after 2 h and totally finished after 5 h. Further studies showed that MCs containing both Arg^2^ and Arg^4^ residues underwent a faster deconjugation process than MCs with only one Arg residue, which in turn underwent a faster deconjugation process than MCs with no Arg residue. Similarly, this method was tested on various other MC conjugates: cys-conjugate of [Dha^7^]MC-LR, MeSH conjugate of [Asp^3^]MC-RY, GSH-conjugate of [Dha^7^]MC-LR, and [Asp3]MC-RY yielded a complete reaction within minutes at pH 10.7 and within a few hours at pH 9.2 [113].

#### 4.2.3. Application to Natural Samples

To our knowledge, Foss et al. [59] reported the only application of basic pH deconjugation to natural samples, namely, cyanobacterial bloom samples and mallard duck (*Anas platyrhynchos*) liver. Two samples of lyophilized bloom material (10 mg each) and two samples of duck liver (50 mg each) were extracted directly into vials with 1 mL of MeOH, 500 µL of Na_2_CO_3_ (0.2 M), and 50 µL of DMSO. The vials were then placed on a rotary mixer at around 20 rpm for 10 days. After a centrifugation step at 14,800 rpm for 5 min, the supernatants were centrifugally filtered (0.45 µm) for 20 min before analysis via multi-hapten ELISA. The total MCs analyzed in this study by oxidation and deconjugation counted three times the free MCs analyzed by Adda ELISA in the same samples. Moreover, the analysis following deconjugation at basic pH allowed the detection of 2.5 times more MCs than an extraction performed by deconjugation at neutral pH. When comparing the results between the two methods for the extraction and analysis of the total MCs, depending on the matrix, one or the other method can detect more MCs. For the samples of lyophilized bloom material, the MMPB LC-MS/MS method yielded almost twice as many total MCs (840 vs. 430 µg/g) than the deconjugation/ELISA multi-hapten method. Conversely, in the two samples of mallard duck liver, the deconjugation/ELISA multi-hapten method detected 26% more MCs than the MMPB LC-MS/MS method.

#### 4.2.4. Discussion on the Deconjugation at Basic pH

Deconjugation at basic pH through the two approaches proposed by Zemskov et al. [115] and Miles [113,114] seems to be a promising method for the analysis of the total forms of MCs. Unlike the MMPB method, this method has the advantage of keeping the identity of the variant intact after the extraction and thus provides a better idea of the distribution of the variants when analyzing natural samples using LC-MS/MS but also avoids the inclusion of nodularins in the analysis as in the MMPB method. Although there is limited experience with this method, its recent application to lyophilized bloom material and duck liver [59] has shown encouraging results and, in some cases, even higher extraction yields than the MMPB method. More knowledge of this method is, of course, necessary to characterize the parameters of this method but also its application to other matrices in order to better understand the potential matrix effects related to this method.

## 5. Quantification of Total MC after Laser Irradiation Desorption

A recent method developed by Shen et al. [120] used laser irradiation desorption (LID) as a means to break the covalent bond between MCs and proteins, including PP1 and PP2A. The tests were conducted during the development of the LID protocol to study the relationship between extraction efficiency and laser power density. Among the criteria studied, the time of exposure of the sample to the laser between 1 to 9 min was compared, and the results indicate that the best exposure time is 5 min. The second criterion studied was the sample volume. An excessive volume would lead to the dilution of the laser energy, while a volume that is too low would lead to an energy that is too high. Five extraction volumes were tested (1, 2, 3, 5, and 8 mL). Similar extraction yields were obtained with volumes of 1, 2, 3, and 5 mL, while a decrease in the yield of 15 to 20% was found with volumes exceeding 8 mL. The volume selected after these tests was 5 mL. The method was validated by studying different parameters such as selectivity, linearity, sensitivity (LOD and LOQ of the method were lower than 0.16 and 0.52 µg/kg, respectively), accuracy, stability, and recovery (relative recoveries of MC ranging from 83.4 to 95.2%). Thus, the optimized protocol corresponds to a 2 g portion of fresh fish tissue (here surimi) crushed and then spiked with 10 µL of a mixture of MCs (MC-RR, -YR, and -LR). The tissues were extracted with 10 mL of MeOH:water:trifluoroacetic acid (TFA) (90:19.9:0.1) and then exposed to visible beam laser irradiation at 450 nm and 8 W for 5 min. After centrifugation at 10,000× *g* for 10 min, the extract was evaporated to 1 mL and analyzed by LC-MS/MS. Once optimized, this method was compared to two other methods: one method in which the tissues were extracted by 4 mL of acidified water at pH = 2 and heated to 80 °C before LC-MS/MS analysis, and another method in which 50–100 ng of MC standard were analyzed by LC-UV after extraction with 75% MeOH and a clean-up of the sample using an immune-affinity column. The LID method (whose recoveries of -RR, -YR, -LR were 90.9%, 88.5%, and 86.3%, respectively) showed better yields than the method using acidified water (whose recoveries of -RR, -YR, -LR were 62%, 76%, and 71%, respectively) but slightly lower yields than the third method on MC standards (whose recoveries of -RR, -YR, -LR were 94%, 92%, and 95%, respectively). However, it should be noted that the third method does not seem to be really comparable to the other two because of the absence of a matrix and, therefore, no possibility of binding of MCs to proteins, which could complicate the analysis. The development of the method having been carried out on samples of fish tissues spiked with MCs, it was necessary to test the LID method on tissues naturally contaminated with cyanobacteria which can contain both free and bound forms of MCs. The LID method was finally tested on 18 fishes from five different species (*Carassius auratus*, *Ctenopharyngodon idellus*, *Aristichthys nobilis*, *Mylopharyngodon piceus*, and *Hypophthalmichthys molitrix*). Almost all of the fish species were MC positive, except *Aristichtys nobilis* and MCs were detected in six different fish and quantified in four with MC values between 1.36 and 6.78 µg/kg in *Ctenopharyngodon idellus* I and *Mylopharyngodon piceus* II, respectively. Yet, there is no information in this study about the total MCs naturally present in the samples, determined using a different method (e.g., Lemieux oxidation) to prove the extraction efficiency of the LID method regarding free and bound MCs. Furthermore, it is possible that during the methodological development, the samples are spiked with MCs but that the reported extraction yields are only related to the free forms of toxins. This method should be compared to another “reference” method for the extraction of the total toxins to demonstrate its ability to extract both free and bound forms of MCs.

## 6. Conclusions

As bound MCs can account for a significant part of the total MCs bioaccumulated in an organism, it is thus necessary to develop and optimize the methods for the extraction and analysis of total MCs. Among the four methods presented in this review (MMPB method, deconjugation at basic pH, ozonolysis, and LID), the MMPB method seems to be the one that has been applied the most for a variety of matrices and for which there have been the most attempts of improvement. Attempts to optimize each step of the MMPB method have been made, for example, during the pre-treatment of the sample, whether chemical or enzymatic, or whether it aims to lyse the cells and concentrate the sample. In the same way, different steps of Lemieux oxidation, extraction/purification, and analysis have evolved, as shown in Table 1, which summarizes the different protocols for the MMPB method implemented in the literature. However, the performances of this method remain very variable, and it is necessary to continue improving key steps, in particular, the purification step, the critical phase of the MMPB method. More recently, deconjugation at basic pH has shown comparable extraction yields to the MMPB method in its first application to cyanobacterial and animal matrices. It would be relevant to compare the different protocols but also to extend the tests to different matrices. The third method reviewed here, ozonolysis, presents good extraction yields for cyanobacteria and sediments but has never been applied to animal matrices where MCs could be bound to proteins. It would therefore be necessary to extend this method to these kinds of matrices. Few studies used ozonolysis, probably due to the specific equipment required to carry out this method. Finally, the LID method is the most recently developed method for the extraction of the total forms of MCs and presents yields higher than 85% on free forms without really demonstrating its efficiency on bound forms. Moreover, it would be necessary to extend it to other matrices, and unlike the MMPB and deconjugation approaches, this method requires specific equipment. Among the different methods reviewed in this article, the MMPB method seems to be the reference despite matrix- and protocol-dependent extraction yields and important matrix effects. Conversely, the other methods reviewed here (deconjugation, ozonolysis, and LID) could be interesting alternatives to the MMPB method but require more hindsight on their use and performance. Although these tests are not yet routinely applied to samples, they would allow a better understanding of the distribution of free/bound toxins, for example, during the accidental intoxication of animals with cyanobacteria, but it would also be a first step towards estimating the sanitary risk during the consumption of species contaminated by MCs.

## Data Availability

Not applicable.

## Supplementary Materials

The following supporting information can be downloaded at: https://www.mdpi.com/article/10.3390/toxins14080550/s1, Table S1: Different genera of cyanobacteria known to produce microcystins classed by environment and morphotype (modified from [1], page 56).
