# Supplementary material for: Analysis of Total-Forms of Cyanotoxins Microcystins in Biological Matrices: A Methodological Review
Source: Toxins (Basel). 2022 Aug 11;14(8):550. doi: 10.3390/toxins14080550 (PMC9416067; doi:10.3390/toxins14080550)
Supplement: Supplementary file 1 [file toxins-14-00550-s001.zip › toxins-1808305-supplementary.pdf]

toxins

Supplementary Materials: Analysis of Total-Forms of Cyanotoxins Microcystins in Biological Matrices: A Methodological Review

Pierre Bouteiller, Emilie Lance, Thierry Guérin and Ronel Biré

Table S1. Different genera of cyanobacteria known to produce microcystins classed by environment and morphotype (modified from [1], page 56).

Environment	Morphotype	Main genera of cyanobacteria proven producers	Other genera of cyanobacteria known producers
Fresh water and terrestrial environment (cyanobacteria in symbiosis with fungi to form lichens), brackish estuarine water.	Unicellular colonial	*Microcystis*	*Aphanocapsa, Merismopedia, Radiocystis, Woronichinia*
	Filamentous	*Planktothrix (Oscillatoria)*	*Annamia, Geitlerinema, Leptolyngbya, Limnothrix, Kamptonema/Phormidium/Microcoleus, Pseudanabaena, Spirulina, Trichodesmium, Plectonema*
	Filamentous with heterocyst	*Anabaena*	*Anabaenopsis, Calothrix, Nostoc, Trichormus*
	Filamentous with heterocyst and branching	*Hapalosiphon*	*Fischerella*

Reference

1. ANSES. *Évaluation des risques liés aux cyanobactéries et leurs toxines dans les eaux douces*; 2020; p. 495.
